# Supplementary material for: Overall Survival of Patients with Myxofibrosarcomas: An Epidemiological Study
Source: Cancers (Basel). 2022 Feb 22;14(5):1102. doi: 10.3390/cancers14051102 (PMC8909833; doi:10.3390/cancers14051102)
Supplement: Supplementary file 1 [file cancers-14-01102-s001.zip › cancers-1589330-supplementary.pdf]

cancers

MDPI

Supplementary data: Overall survival of patients with myxofibrosarcomas: an epidemiological study

Table S1. Clinical characteristics and OS.

Variable	No. (%)	Median Overall survival (months) (95% CI[1])	p-value[2]
**Age**			
<65 years	395 (44)	Median not reached	<0.01[3]
≥65 years	513 (56)	80.1 (63.9-96.3)	
**Sex**			
Male	492 (54)	133.9 (106.5-161.2)	0.025[3]
Female	416 (46)	194.1 (146.2-241.9)	
**Cancer in medical history**			
No	766 (84)	161.8	<0.01[3]
Yes	142 (16)	59.2 (40.2-78.2)	
**Tumour size**			
≤5 cm	375 (41)	Median not reached	<0.01[3]
>5 cm	498 (55)	89.1 (64.0-114.2)	
Unknown	35 (4)	126.5 (56.1-196.8)	
**Tumour depth**			
Superficial	420 (46)	Median not reached	<0.01[3]
Deep	267 (29)	105.5 (73.3-137.7)	
Unknown	221 (24)	126.5 (54.2-198.8)	
**Tumour location**			
Trunk	183 (20)	133.9 (88.0-179.8)	0.19
Head & neck	24 (3)	192.4 (102.9-282.0)	
Extremities	686 (76)	156.6 (126.1-187.2)	
(Retro)peritoneum	5 (0.5)	20.1 (0.0-51.2)	
Other	10 (1)	67.543	
**Histological grade**			
I	241 (27)	Median not reached	<0.01[3]
II	188 (21)	112.9 (76.3-149.6)	
III	358 (39)	89.1 (59.6-118.6)	
Unknown	121 (13)	133.9 (50.9-216.9)	
**Distant metastasis at diagnosis**			
No	791 (87)	150.2 (115.2-185.1)	<0.01[3]
Yes	42 (5)	12.2 (7.3-17.0)	
Unknown	75 (8)	Median not reached	
**Surgery**			
No	71 (8)	12.3 (8.2-16.5)	<0.01[3]
Yes	837 (92)	162.3 (140.3-184.3)	
**Residual disease following primary treatment**			
R0	539 (59)	Median not reached	<0.01[3]
R1	164 (18)	104.4 (81.4-127.4)	
R2	13 (1)	50.6 (36.3-65.0)	
Unknown/no surgery	192 (21)	77.1 (11.1-143.1)	
**Radiotherapy**			
No	382 (42)	160.4	<0.01[3]
Neoadjuvant	188 (21)	116.1 (95.8-136.4)	
Adjuvant	291 (32)	161.8 (132.7-190.8)	
Neoadjuvant + adjuvant	19 (2)	Median not reached	
Primary	28 (3)	20.6 (4.4-36.9)	

**Diagnosis**			
Sarcoma centre	183 (20)	194.1 (96.6-291.5)	0.15
Other	725 (80)	146.8 (124.8-168.8)	
**Surgery**			
Sarcoma centre	412 (45)	156.8 (135.4-178.2)	0.02^{3}
Other	480 (53)	126.0 (95.1-156.8)	
Unknown	16 (2)	192.4	

[1] CI (confidence interval) [2] log-rank test [3] $p<0.05$.

Table S2. Patient and clinical characteristics of the 177 patients whose recurrence status was available.

Variables	No. (%)
**Age**	
Median age (range)	66 (22-92)
**Sex**	
Male	88 (50)
Female	89 (50)
**Cancer in medical history**	
No	152 (86)
Yes	25 (14)
**Tumour size**	
≤5 cm	77 (44)
>5 cm	93 (52)
Unknown	7 (4)
**Tumour depth**	
Superficial	92 (52)
Deep	53 (30)
Unknown	32 (18)
**Myxofibrosarcoma localization**	
Trunk	30 (17)
Head and neck	4 (2)
Extremities	140 (79)
Mediastinum/heart/pleura	1 (0.6)
Specific organ	2 (1)
**Sarcoma grading**	
I	51 (29)
II	32 (18)
III	70 (39)
Unknown	24 (14)
**Distant metastases at diagnosis**	
No	165 (93)
Yes	5 (3)
Unknown	7 (4)
**Surgery**	
No	8 (5)
Yes	169 (96)
**Residual disease following primary treatment**	
R0	105 (59)
R1	40 (23)
R2	2 (1)
RX	30 (17)
**Radiotherapy**	
No	94 (53)

Neoadjuvant	17 (10)
Adjuvant	61 (34)
Neoadjuvant + adjuvant	3 (2)
Primary	2 (1)

Table S3. Clinical characteristics and local recurrence and distant metastasis free survival of the 177 patients whose recurrence status was available.

Variables	No. (%)	Median Local Recurrence free survival (months) (95% CI[1])	p-value[2]	Median Distant metastasis free survival (months) (95% CI[1])	p-value[2]
**Age**					
<65 years	78 (44)	194.1 (1.9-386.2)	<0.01[3]	194.054	<0.01[3]
≥65 years	99 (56)	25.1 (17.3-32.9)		40.8 (31.9-49.7)	
**Sex**					
Male	88 (50)	34.4 (27.0-41.8)	0.12	58 (33.0-83.4)	0.194
Female	89 (50)	53.9 (17.4-90.4)		110.5 (64.7-156.3)	
**Cancer in medical history**			0.368		0.232
No	152 (86)	37.8 (28.6-47.1)		84.9 (35.5-134.3)	
Yes	25 (14)	41.4 (33.6-49.2)		40.8 (34.2-47.4)	
**Tumour size**					
≤5 cm	77 (44)	85.8 (34.6-137.0)	<0.01[3]	194.1 (92.3-295.8)	<0.01[3]
>5 cm	93 (52)	34.0 (23.6-44.3)		39.8 (24.2-55.5)	
Unknown	7 (4)	44.8 (0-135.3)		37.0 (0.0-75.5)	
**Tumour depth**					
Superficial	92 (52)	38.9 (30.6-47.3)	0.778	103.5 (57.2-149.9)	0.060
Deep	53 (30)	36.7 (14.6-58.9)		39.8 (13.6-66.0)	
Unknown	32 (18)	44.8 (19.7-69.7)		56.5 (0.0-187.1)	
**Myxofibrosarcoma localization**					
Trunk	30 (17)	25.1 (8.4-41.8)	0.106	43.1 (32.7-53.5)	0.443
Extremities	140 (79)	41.4 (26.1-56.7)		77.4 (41.8-113.0)	
Other	7 (4)	Median not reached		Median not reached	
**Sarcoma grading**					
I	51 (29)	Median not reached	<0.01[3]	Median not reached	<0.01[3]
II	32 (18)	34.4 (13.5-55.3)		43.1 (0.0-96.9)	
III	70 (39)	22.4 (17.0-27.9)		23.4 (14.4-32.3)	
Unknown	24 (14)	50.1 (27.6-72.5)		155.0	
**Distant metastases at diagnosis**					
No	165 (93)	41.2 (29.5-53.0)	0.044	85.2 (40.5-129.9)	0.044
Yes	5 (3)	41.7 (0.0-114.4)		15.1 (0.0-30.8)	
Unknown	7 (4)	17.3 (5.7-28.8)		39.3 (0.0-134.4)	
**Surgery**					
No	8 (5)	9.5 (2.9-16.2)	0.023	13.3 (0.0-38.3)	<0.01[3]
Yes	169 (96)	41.4 (30.1-52.7)		85.2 (42.4-127.9)	
**Residual disease following primary treatment**			0.108		0.072
R0	105 (59)	47.635 (19.6-75.7)		85.2	
R1/R2	42 (24)	22.8 (20.7-47.6)		50.1 (26.7-73.5)	
RX	30 (17)	40.3 (22.5-58.1)		162.3 (67.8-256.8)	
**Radiotherapy**					
No	94 (53)	41.4 (27.1-55.7)	<0.01[3]	Median not reached	<0.01[3]
Neoadjuvant	17 (10)	22.4 (4.7-40.2)		23.4 (10.2-36.5)	
Adjuvant	61 (34)	38.9 (24.5-53.4)		41.2 (20.1-62.2)	

Neoadjuvant + adjuvant	3 (2)	Median not reached	Median not reached
Primary	2 (1)	2.6	2.6

[1] CI (confidence interval) [2] log-rank test [3] $p<0.05$.

Table S4. Multivariable analysis on local recurrence and distant metastasis free survival with imputed data.

	Local Recurrence free survival			*Distant metastasis free survival*		
**Variables**	**Hazard Ratio**	**CI (95%)[1]**	**p-value[2]**	**Hazard Ratio**	**CI (95%)[1]**	**p-value[2]**
**Age**						
<65 years	Ref.	Ref.		Ref.	Ref.	
≥65 years	2.4	1.6-3.8	<0.01[3]	2.7	1.6-4.5	<0.01[3]
**Tumour size**						
<5 cm	Ref.	Ref.		Ref.	Ref.	
≥5 cm	2.1	1.4-3.3	<0.01[3]	2.6	1.6-4.2	<0.01[3]
**Histological grade**						
I	Ref.	Ref.		Ref.	Ref.	
II	2.5	1.2-5.1	0.01[3]	3.5	1.6-7.6	<0.01[3]
III	3.2	1.4-7.5	0.01[3]	4.6	2.2-10.5	<0.01[3]
**Radiotherapy**						
No	Ref.	Ref.		Ref.	Ref.	
Neoadjuvant	0.5	0.2-1.0	0.045[3]	1.1	0.5-2.3	0.79
Adjuvant	0.4	0.2-0.7	<0.01[3]	0.7	0.4-1.3	0.25

[1] CI (confidence interval) [2] log-rank test [3] $p<0.05$.
